# Spontaneous Recovery in Complete Foot Drop in a Case of Lumbar Disc Herniation: A Neurological Surprise

**DOI:** 10.7759/cureus.20962

**Published:** 2022-01-05

**Authors:** Abhijit Ravindra Chandankhede, Dhruv Talwar, Sourya Acharya, Sunil Kumar

**Affiliations:** 1 Department of Neurosurgery, Jawaharlal Nehru Medical College, Datta Meghe Institute of Medical Sciences (Deemed to be University), Wardha, IND; 2 Department of Medicine, Jawaharlal Nehru Medical College, Datta Meghe Institute of Medical Sciences (Deemed to be University), Wardha, IND

**Keywords:** degeneration of disc, neurological impairment, spontaneous recovery, foot drop, lumbar intervertebral disc prolapse

## Abstract

Lumbar intervertebral disc prolapse has been associated with radiculopathy and the sensory and motor changes that occur as the result of neural compression. The most important motor symptom is foot drop. Occurrence of foot drop in lumber protrusion of intervertebral disc prompts for the surgical treatment of the condition. Here, we report a case of a 32-year-male presented with unilateral foot drop, diagnosed as lumbar protrusion of intervertebral disc and recovered significantly without surgery. The good neurological outcome of the conservative management, in this case, puts the surgeon in a quandary whether to offer surgical management or not. A clinician should always remember this outcome before choosing the management plan for lumbar protrusion of intervertebral disc although rare.

## Introduction

Foot drop is the inability to dorsiflex the foot and the toes. The neurological impairment causing the foot drop can be central or peripheral [1]. The central pathologies include motor neuron disease, parasagittal cortical or cerebral subcortical lesions [2]. The peripheral causes can be lumbar radiculopathy or mononeuropathies of sciatic, deep peroneal, or common peroneal nerves.

Lumbar degenerative discs and herniation of the L4-L5 intervertebral disc have been commonly implicated as the cause of unilateral foot drop [3]. Many studies have stated the role of lumbar protrusion of the intervertebral disc in causing foot drop. Here, we report a case of spontaneous recovery of unilateral foot drop in a case of lumbar protrusion of intervertebral disc within 10 days of conservative management with significant (70-75%) improvement in the low backache and foot drop.

## Case presentation

**Figure 1:**
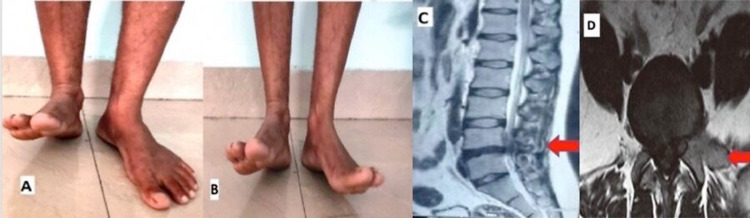
(A) Clinical photograph at the first visit showing left foot drop and (B) at follow up visit showing significant improvement of foot drop. (C) Midsagittal MRI lumbar spine showing L4-L5 PIVD and (D) axial MRI lumbar spine showing protruded disc with foraminal compression PIVD: prolapsed intervertebral disc

A 32-year-male presented to the outpatient department with complaints of insidious onset low backache for 15 days. The pain was radiating to the left lower limb on the posterior aspect getting worse on bending forward and climbing stairs. He took oral analgesics and rest for five days, during which pain was significantly reduced. However, after five days, he was unable to lift the left foot above the ground level and he needed to take higher steps on the left side while walking. He didn’t have any history of fall or trauma. On neurological evaluation, he had a complete foot drop on the left side (MRC 0/5), also weakness in the hip abduction and knee flexion (MRC 4/5) as shown in Figure 1, panel A (later resolved as shown in Figure 1, panel B). There were no sensory loss and no bowel and bladder involvement. Deep tendon reflexes were normal in both the lower limbs and the straight leg raising test was positive on the left side reproducing the patient’s radiating pain symptoms. The passive movements of both the feet were normal and full range of motion was present without any tenderness. Other movements and range of motion at the knee and hip were normal.

He was evaluated with lumbosacral magnetic resonance imaging (Figure 1, panels C and D). There was degenerated L4-L5 disc protrusion with left foraminal compression. In view of the symptomatology and imaging findings, he was diagnosed to have L4-L5 intervertebral disc degeneration and protrusion with L5 radiculopathy and complete foot drop on left. The patient denied surgical intervention and was managed on conservative management with medications and physical therapy. Medical treatment in the form of muscle relaxant (chlorzoxazone 500 mg twice a day) and non-steroidal anti-inflammatory drug (ibuprofen 400 mg twice a day) was prescribed and the patient was called for a follow-up.

He was reviewed at the outpatient department 10 days later, and there was a significant recovery of the left foot drop (grade 4/5) and the patient was able to walk without high stepping of the left lower limb. Due to the significant improvement of the foot drop and reduction in the backache, it was then decided to continue conservative management and to do surgical intervention only if any further neurological deterioration occurs. On the second follow-up after three weeks, the patient regained normal strength of the dorsiflexion (power 5/5) and the gait was normal (Figure 1, panel B).

## Discussion

Foot drop can be the result of either muscular or neurological disorders. A retrospective study of 303 patients of foot drop by Van Langenhove et al. recognized causes to be of central neurogenic origin in 31% and of peripheral neurogenic origin in 68% of patients. The peripheral causes were common peroneal nerve lesions (30.6%), L5-radiculopathies (19.7%), and polyneuropathies (18.3%). Also, most of the peripheral origin foot drop was of traumatic origin [4].

The common peroneal nerve lesion leads to involvement of only everters and dorsiflexors of the foot while L5 nerve root involvement causes weakness of hip abductors and knee flexors as occurred in our case, thus clinically localizing the causes of foot drop to the L5 nerve root in our case. The L5 nerve root is involved in the protrusion of lumbar intervertebral disc at L4-L5 level in most of the cases as described by a systematic review by Wang and Nataraj [5]. The L5 root can be involved due to a paracentral bulge of the intervertebral disc at L4- L5 level or due to lateral protrusion of the disc at the L5-S1 level. A few cases of lumbar canal stenosis associated with foot drop have also been described [6].

Bilateral foot drop can be seen in central lumbar protrusion of intervertebral disc; it may be sudden onset or slow in nature. Several articles describe this phenomenon reporting the foot drop improving after surgery for lumbar protrusion of intervertebral disc or lumbar canal stenosis [7,8]

The recovery of foot drop in cases of lumbar protrusion of intervertebral disc has been a topic of discussion for long. A study by Takenaka and Aono recognizes that the weaker the muscle power, the less are the chances of neurological recovery at the two years follow-up [9]. Other predictor identified is the duration of the foot drop before the surgery. The indirect predictors in this study were age at surgery, presence of leg pain, and herniated or non-herniated lumbar disc (Table 1). Similar results have been interpreted by the study by Bhargava et al. stating that the weaker the anterior tibialis muscle power and the longer the duration of the foot drop, the less is the recovery of the foot drop even after successful surgical intervention for lumbar protrusion of intervertebral disc [10].

**Table 1:** Factors influencing outcome of foot drop PIVD: protrusion of intervertebral disc; UMN: upper motor neuron; LMN: lower motor neuron

S. no	Factors
1	Cause of foot drop - UMN or LMN
2	Power of tibialis anterior on presentation
3	Duration of foot drop
4	Age of patient at surgery
5	Presence of leg pain
6	Location of disc - central or lateral
7	Single disc vs multiple PIVD

None of the above-quoted studies have included non-operative patients for evaluation of the recovery of tibialis anterior muscle strength in patients with lumbar protrusion of intervertebral disc. Upon detailed literature search, we observed only one such case reporting the similar surprising phenomenon of spontaneous recovery of complete foot drop in lumbar protrusion of intervertebral disc [11]. Although the duration of foot drop was shorter in this case indicating no permanent damage to the nerve root.

The paucity of reporting in the literature of this wonderful phenomenon of spontaneous recovery in the complete foot drop without any surgical intervention makes this a unique case to report and discuss. The patient is being followed regularly for any change in the neurological status and does not have any deterioration in muscle power so far.

It is noticed that the acute foot drop in lumbar protrusion of intervertebral disc needs early surgery, which is not always correct. A study conducted by Albayrak et al. reported that late presentation to surgery had poor outcomes in cases of foot drop with lumbar disc pathology [12].

In contrast, we postulate that, given a chance, anti-inflammatory drugs may sometimes reduce edema and may be helpful to the patient. Especially, when there is nerve root compression, edema plays an important role in the pathophysiology. Further studies are needed to study the incidence and outcome of lumbar disc herniation patients who are managed with conservative management.

## Conclusions

The importance of detailed clinical examination and history taking cannot be overemphasized. The positive predictors of spontaneous recovery like age, anterior tibialis muscle power, duration of foot drop, the reason for the foot drop (upper motor neuron or lower motor neuron), absence of the urinary complaints point towards the chance of such spontaneous recovery. Sequential neurological examination remains the cornerstone in the planning of the surgical or conservative management of the case depending on the clinical findings and patient factors. If the patient is improving on anti-inflammatory drugs, the patient can be observed for seven to 10 days and if not improving, may be considered for surgery.
